# Implementing a paediatric early warning score into pre-hospital practice

**DOI:** 10.29045/14784726.2019.06.4.1.42

**Published:** 2019-06-01

**Authors:** Martin Rolls

**Affiliations:** North West Ambulance Service

## Abstract

**Aim:**

This study addressed a desire by ambulance clinicians for additional education in the examination and assessment of the unwell child; it also explored whether ambulance clinicians could use a paediatric early warning score (PEWS) safely and effectively in the pre-hospital arena.

**Methods:**

A small-scale study introduced a validated PEWS into pre-hospital practice. The paediatric observations priority score (POPS) combines physiological observations with clinicians’ review. POPS uses a range of proxy measures such as work of breathing, alertness, gut feeling and known high-risk factors, to further refine the scoring. Based on a sample of over 24,000 patients, POPS has been validated for use in emergency departments (EDs). POPS can identify potentially critically unwell children as well as those fit for discharge without hospital admission, the fundamental purpose of an ED.

Study participants were surveyed before and after the trial period in order to examine self-reported scores in confidence and competence levels for the child in pain, the breathless child, the child with a decreased level of consciousness, the febrile child and the seriously injured child.

Completed patient report forms (PRFs) were returned to the principal investigator for further analysis. PRFs were re-distributed among participants for rescoring. Once rescoring was completed, the PRFs were returned to the principal investigator for calculation of interrater reliability. Participants remained anonymous for the survey.

**Results:**

Interrater reliability (Kappa coefficient) was calculated as 0.401, which is considered moderate agreement. As POPS rose, variance decreased. Lower POPS had variance, but these patients were lower acuity. Equal scoring in the main was reliable.

**Conclusion:**

For a cohort of ambulance clinicians, POPS was found to be safe and effective. Self-reported levels in confidence and competence improved in all patient presentations when comparing before and after the trial period (Table 1).

**Table 1.** Comparison of mean scores for confidence and competence before and after trial period, stratified by patient presentation.

	Comparison of mean scores					
	Confidence			Competence		
	Before	After	Diff (+/-)	Before	After	Diff (+/-)
**Pain**	5.01	6.34	1.33	4.17	7.49	3.32
**Breathless**	5.13	6.52	1.39	6.54	7.62	1.08
**Decreased level of consciousness**	5.93	6.47	0.54	6.04	7.58	1.54
**Febrile**	6.92	7.06	0.14	6.85	8.20	1.35
**Seriously injured**	5.95	6.44	0.49	5.99	7.60	1.61

